# In Situ Image Acquisition and Measurement of Microdroplets Based on Delay Triggering

**DOI:** 10.3390/mi10020148

**Published:** 2019-02-22

**Authors:** Xuefeng Chang, Kang Zheng, Dan Xie, Xiayun Shu, Keyu Xu, Wenhuan Chen, Bo Li, Changjian Wu

**Affiliations:** 1 School of Mechanical and Automotive Engineering, Xiamen University of Technology, Xiamen 361024, China; xfchang@xmut.edu.cn (X.C.); xukeyu@stu.xmut.edu.cn (K.X.); chenwenhuan@stu.xmut.edu.cn (W.C.); libo@stu.xmut.edu.cn (B.L.); wuchangjian@stu.xmut.edu.cn (C.W.); 2 Key Laboratory of Precision Actuation and Transmission, Fujian Province University, Xiamen 361024, China; zhengkang163@163.com; 3 Program for Innovative Research Team in Science and Technology in Fujian Province University, Fujian Province University, Xiamen 361024, China

**Keywords:** microdroplet jetting, in situ, image processing, delay triggering

## Abstract

An in situ image acquisition apparatus based on delay triggering for visualizing microdroplets formation is described. The imaging system includes a charge-coupled device camera, a motion control card, a driving circuit, a time delay triggering circuit, and a light source. By adjusting the varying trigger delay time which is synchronized with respect to the signal for jetting, the steady sequential images of the droplet flying in free space can be captured real-time by the system. Several image processing steps are taken to measure the diameters and coordinates of the droplets. Also, the jetting speeds can be calculated according to the delay time interval. For glycerin/water (60:40, mass ratio), under the given conditions of the self-made pneumatically diaphragm-driven drop-on-demand inkjet apparatus, the average of diameter and volume are measured as 266.8 μm and 9944 pL, respectively, and the maximum average velocity of the microdroplets is 0.689 m/s. Finally, the imaging system is applied to measure the volume of 200 microsolder balls generated from the inkjet apparatus. The average diameter is 87.96 μm, and the relative standard deviation is 0.83%. The results show good reproducibility. Unlike previous stroboscopic techniques, the present in situ imaging system which is absence of instantaneous high intensity light employs two control signals to stimulate the microdroplet generator and the charge-coupled device (CCD) camera. Hence, the system can avoid the desynchronization problem of signals which control the strobe light-emitting diode (LED) light source and the camera in previous equipment. This technology is a reliable and cost-effective approach for capturing and measuring microdroplets.

## 1. Introduction

Drop-on-demand microjetting is a noncontact printing method for depositing microdroplets on various substrates. This technology has been widely used in a variety of applications including solders, ceramics, polymers, biopolymers arrays, and so on [1,2]. In order to be a reliable production technology, the jetting performance should be captured and controlled accurately. Then, the approach to obtain the sequential images of the microdroplet formation process and measure the droplet characteristics including size and speed are two essential crucial factors. However, the whole stages during drop formation last less than 3 ms. In this process, even a 104 FPS (frames per second) camera can capture only one or two images. Therefore, the common high-speed camera simply cannot capture the details of the microdroplet formation processes. The ultrahigh-speed camera can directly implement sequential photograph for jetting process. But the price is extremely expensive [3,4]. Accordingly, a reliable and low-cost image system for visualizing the microdroplet formation dynamic motion is necessary.

In recent years, the emergence of stroboscopic technique provided an effective approach to record microjetting. Along with this technology was put forward, many image acquisition systems of microdroplets based on delay triggering have been proposed, and their components and features are illustrated in Table 1. Dong et al. [5] described a visualizing apparatus based on flash photography including a pulsed laser, a charge-coupled camera, and signal generators. Sharp images with sufficient temporal resolution had been obtained. Hutchings et al. [6] described a technique which had been developed for analyzing the shape and development of ink jets and drops. Kwon et al. [7] proposed an image measurement system to observe and analyze the image of the droplet. The components were the multifunction I/O unit, a strobe light-emitting diode (LED) light and a charge-coupled device (CCD). The two counters in multifunction I/O unit could generate two digital pulse trains. The first digital pulse was to stimulate the waveform for the jetting. The second was used as a trigger signal for controlling a strobe LED light which was synchronized with the signal used to produce the ink jet. By adjusting the delay time, the microdrop formation process could be obtained. The in situ measurement system built by Fan et al. [8] was composed of a CCD camera, a zoom lens, and a stroboscopic light. The strobe light was simultaneously triggered by the same frequency as the function generator which actuated the piezoelectric ceramic film. By selecting a proper shutter speed, a steady image of the droplet could clearly be viewed and captured by the camera. Jo et al. [9] also employed the stroboscopic method to get the pictures of drop deformation by changing the delay time between the pulse inception and the flash of LED light. The precise real-time measurement technology proposed by Thurow et al. [10] was implemented in most appliances with the aid of a stroboscope camera system. The hardware was made up of a camera with an enlarging lens and a LED stroboscope. In the international standards of IEC 62899-302-1:2017 Printed electronics Part 302-1 and IEC 62899-302-2:2018 Printed electronics Part 302-2, the imaging based measurement of jetted drop speeds and drop volume was described [11,12]. By means of the above techniques, the speed and diameter of the microdroplet could be calculated based on the CCD camera image and the delay time [13,14,15]. Martin et al. published analysis of inkjet droplet image processing techniques and errors [16,17]. Laser holographic methods in 3D are described by Martin et al. [18] Hsiao et al. has published imaging work on solder balls [19]. Jung described a high-speed imaging which was used to analyze the impact and spreading the drops onto smooth glass surfaces [20]. Jiang et al. used a low-cost microcontroller board (typical cost is less than a hundred US dollars) as the pulse generator in a flash photographic system [21]. Martin et al. also described the measurement of the volumes of small (10–100 µm) liquid drops is important in a number of fields including inkjet printing, liquid dispensing, and spraying [22].

Among these systems, the print heads are all piezoelectric transducers and the image systems include the stroboscopic illumination source and CCD camera. Then, three control signals are applied to operate the ejection of the microdroplet, fire the strobe, and trigger the CCD. Therefore, the camera exposure moment must strictly coincide with the firing time of the LED light because photographing needs instantaneous high intensity light. Otherwise, the microdroplet image will be blurred due to insufficient illumination. However, the desynchronization problem about the signals will inevitably affect measurement quality and accuracy. Consequently, reducing the number of control signals and employing continuous intense light source of stable luminous flux are ideal techniques to improve the reliability and precision of this type of low-cost image system.

This paper proposed a novel in situ image acquisition and measurement of microdroplets based on delay triggering. Using a high-speed CCD camera, a motion control card, a driving circuit, a time delay triggering circuit, and a light source, an imaging system was built to obtain the sequential images of microdroplets generated through the pneumatically diaphragm-driven actuator. By adjusting the delay time, stable microdroplet fabrication process could be captured. The image processing methods have been adapted to measure the diameters, coordinates, and speeds of the microdroplets in real-time.

## 2. Experimental Set-Up

### 2.1. Pneumatically Diaphragm-Driven Actuator

**Figure 1:**
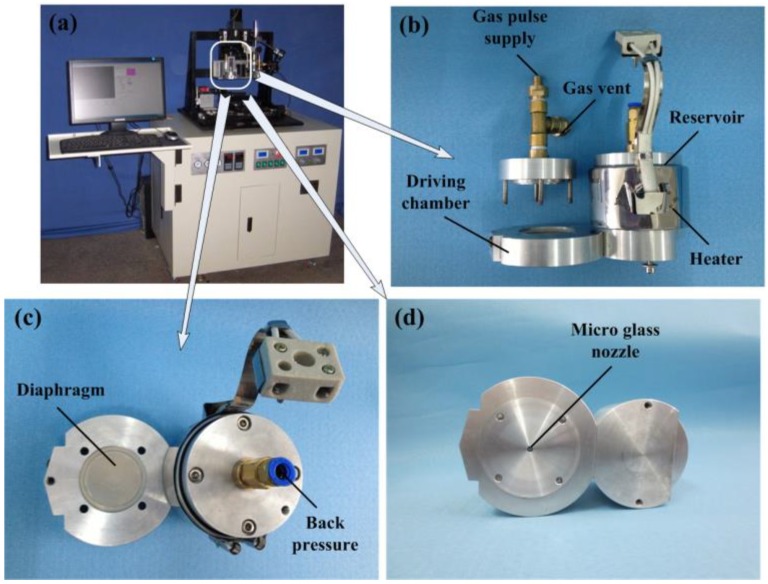
(**a**) Drop-on-demand microjetting system, (**b**) side view, (**c**) top view, and (**d**) bottom view of pneumatic diaphragm microdroplet actuator.

Figure 1 shows photos of drop-on-demand microjetting system and pneumatic diaphragm microdroplet actuator. The actuator consists of a reservoir, a pneumatic chamber connecting with a solenoid valve, a driving chamber separated by a diaphragm made of stainless steel, and a microglass nozzle. When a voltage pulse is applied to the solenoid valve, the generated pneumatic pressure pulse will act on the diaphragm. Then, the diaphragm will bend and its deformation will push a certain amount of liquid out of the nozzle and form the microdroplet.

### 2.2. In Situ Imaging System

**Figure 2:**
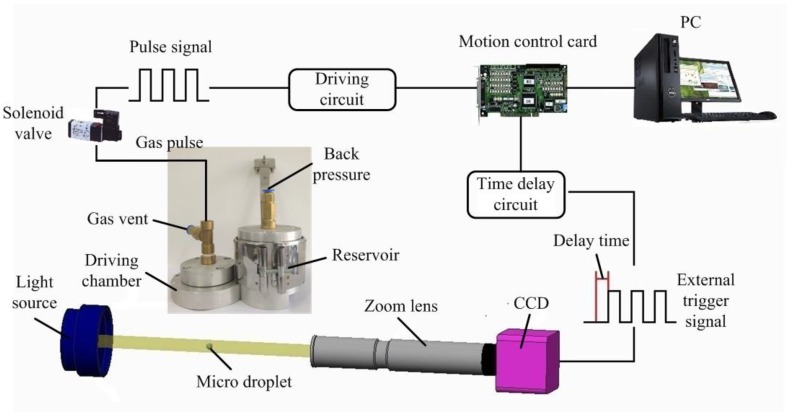
In situ image acquisition and measurement system.

Figure 2 shows the in situ imaging system setup used for observing the microdroplet performance. The system is composed of a high-speed CCD camera, a 4× zoom lens, a light source with the accessory of the fiber light guide, a motion control card, a driving circuit, and a time delay circuit. The Volpi Intralux series light source provides a reliable, dependable, high-intensity cold halogen light. The Intralux includes infrared reflecting filters to ensure the coldest light and the unique crescent shaped diaphragm, which provides constant color temperature, light-emitting angle, and continuous intensity adjustment. The selected model is IX 4000-1, and the specifications are shown in Table 2. The external trigger signal is simultaneously stimulated by the same frequency as the generator which actuates the solenoid valve. Although the measured microdroplets are fast moving objects, a steady image of the microdroplet can clearly be captured by the system. When the time of exposure is too long, the image edge will be blurred. The time of CCD exposure has been set to 35 μs, and the brightness and the contrast are both adjustable on demand.

**Figure 3:**
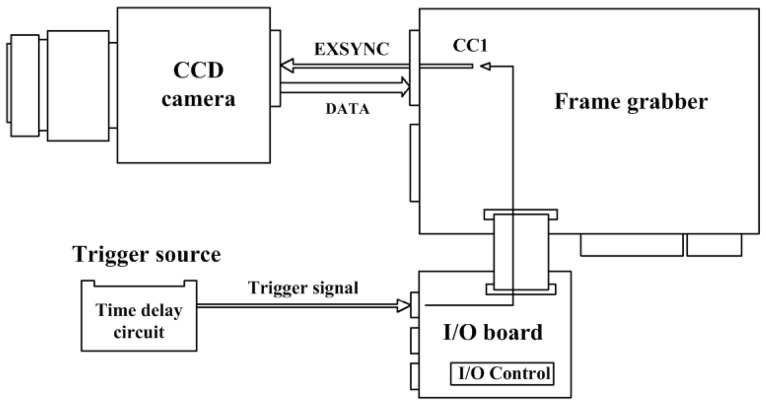
Trigger delay visualization from the trigger source to the camera.

For the CCD camera, a Photonfocus MV-D750E with resolution of 1024 × 760 pixels is used. The camera includes free running and external trigger mode. When the acquisition of an image needs to be synchronized to an external event, a trigger mode can be used. As seen in Figure 3, camera control signals (CC-signals) can be defined by the camera manufacturer to provide certain signals to the camera. There are four CC-signals available and are all unidirectional with data flowing from the frame grabber to the camera. For example, the external trigger is provided by a CC-signal.

**Figure 4:**
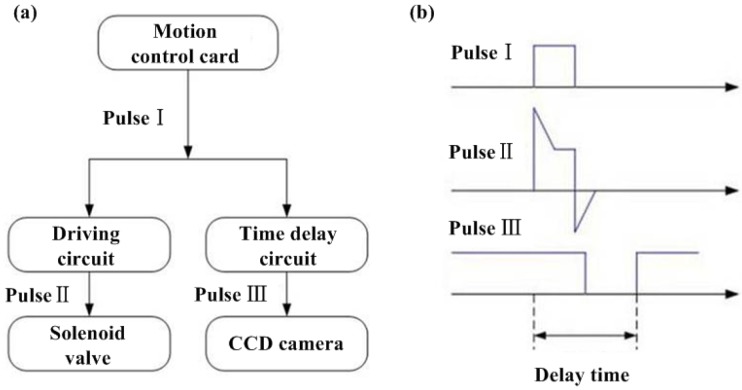
(**a**) Pulse signal flow chart, (**b**) pulse signal timing sequence diagram.

**Figure 5:**
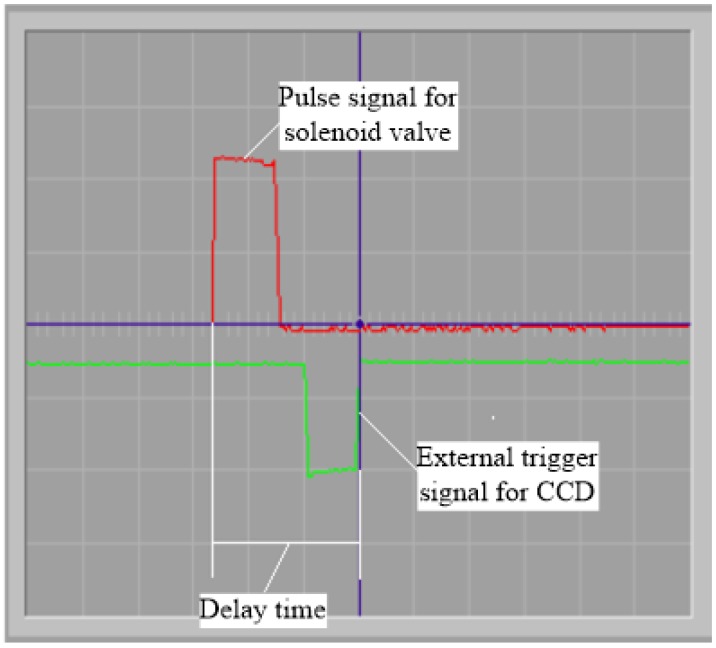
Pulse signal for solenoid valve and external trigger signal for charge-coupled device (CCD).

**Figure 6:**
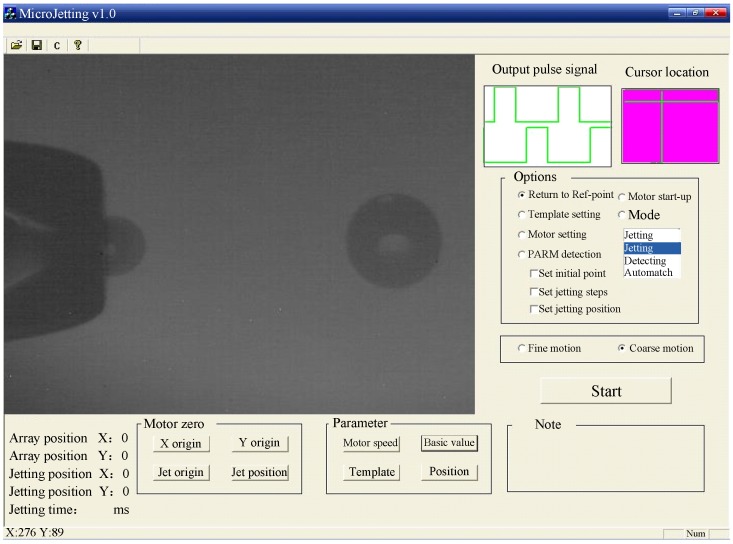
Operation interface of microjetting system.

In this work, the motion control card and the time delay circuit are used to generate digital pulse trains as Figure 4 shown. Pulse I is generated from motion card. Pulse II is used as pulse signal for solenoid valve in order to generate the waveform for the jetting. Pulse III is used as external trigger signal for controlling CCD. The delay time can the modulated manually via an adjustable resistance in the time delay circuit. Due to the time delay in Pulse III, the droplet at the delayed time can appear to be steady. When setting a certain minimal interval time step, we can get the combination pictures of the ejecting process. Because the ejecting process pictures are captured at different times, reproducibility is the prerequisite for the technology. By the imaging system, the ejected trajectory can be captured and the droplet diameters and velocities can be measured from these pictures. The actual time delays between the drop ejection pulses and the external trigger pulses can be captured with an oscilloscope, as Figure 5 shown. Figure 6 is the operation interface of microjetting system developed by Visual C++ 6.0. The camera image can be imported into the software using the frame grabber.

## 3. Image Acquisition

**Figure 7:**
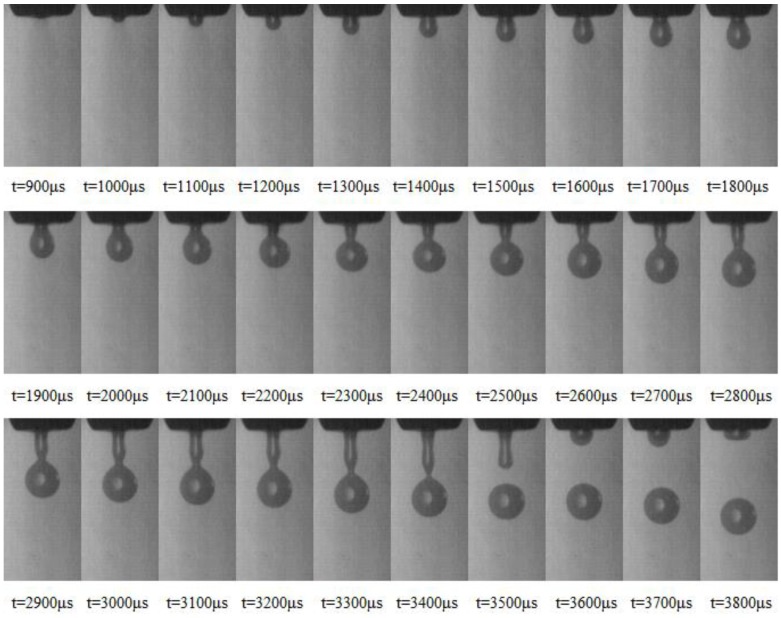
Time-lapse images of microdroplet generation (one primary droplet).

Time-lapse images of microdroplet generation can be easily acquired by the in situ imaging system. When setting the minimal interval time step as 100 μs, though adjusting the variable resistance in the time delay circuit, we can get the combination pictures of the ejecting process by adjusting the interval from 0 to 5000 μs or more. In our experiment, 50 samples of the microdroplet are captured and evaluated at each moment. And 5 sets of sequence diagram are obtained to illustrate the flight and formation process of microdroplets, as the Supplementary Materials shown. Figure 7 describes the time-lapse images of microdroplet generation with one primary droplet. The solution is glycerin/water (60:40, mass ratio). The viscosity and surface tension are 10 MPa·s and 69 mN/m, respectively. When the driving pressure was 0.39 Mpa, the time of high level for input pulse was 1.7 ms and the operating frequency was 2 Hz; the ejecting process from 900 μs to 3800 μs is shown in Figure 7. It can be clearly seen the steps of ejection, elongation, necking, breakup, oscillation, and formation; only one primary droplet came into being under these conditions. Since the ejected trajectory can be captured, the droplet diameters and velocities can be measured from these pictures through the method of image processing.

## 4. Image Processing

The digital image signals captured directly by CCD generally contain a variety of noise and distortion. The typical defects include gray concentration caused by insufficient illumination and noisy signal during the transmission process from CCD to the computer. Although the improvement of image capture hardware can effectively reduce the defects, image processing technology on the initial image for correction, enhancement, noise reduction, and so on, it is an essential way to get a distinct image and obtain the accurate data. The image processing algorithms and extraction methods of microdroplet are described as follows.

### 4.1. Pretreatment of Droplet Images

Because the collected original images have salt-and-pepper noise, and the target (droplet) and background contrast are not high, the acquired image needs to be enhanced. The purpose of image enhancement is as follows. Firstly, the visual effect of the image and the clarity of the image can be improved. Secondly, the image can be converted into a more suitable form for human or machine analysis and processing. According to the purpose and effect of enhancement, there are many methods for image enhancement. Image enhancement can be divided into grayscale correction, image smoothing, image sharpening, image enhancement, and color enhancement according to processing space. The median filtering method is used to preprocess the collected images. The reasons why the median filtering method is an appropriate method are as follows.

(1) Median filtering is a typical nonlinear filtering technique, which can overcome the image detail blur caused by linear filters (such as the mean filter) under certain conditions.

(2) Median filter is a typical low-pass filter, which is mainly used to suppress impulse noise. It can completely filter out the interference noise of sharp wave, and at the same time has the characteristics of better protection of the edge of the target image.

(3) The median filter is nonlinear and has a strong filtering effect on salt-and-pepper noise or pulsed interference.

The three characteristics of the median filter method are exactly in line with the requirements of “protecting the edge of the target image and eliminating salt-and-pepper noise”. The image preprocessing achieved good results. Subsequently, in order to further the contrast between the droplet and the background, a grayscale linear transformation was performed on the median-filtered picture. Finally, the small area where the droplets are located is obtained by means of threshold segmentation, feature extraction, and morphology.

**Figure 8:**
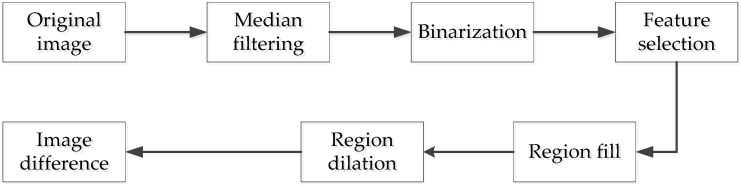
Image preprocessing flow chart.

**Figure 9:**
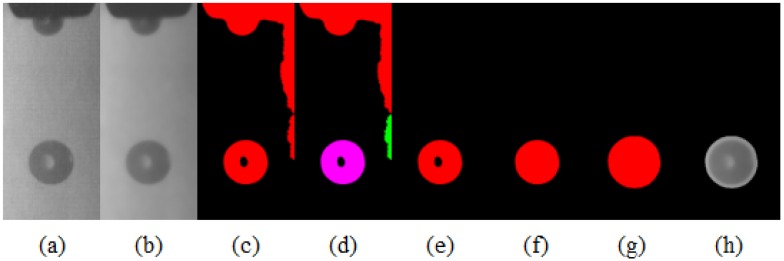
Droplet image preprocessing. (**a**) original image, (**b**) median filter, (**c**) binarization, (**d**) connection, (**e**) feature selection, (**f**) region fill, (**g**) region dilation, and (**h**) image difference.

The purpose of the above method is to reduce the scope of image processing to a range of radius (R + Δr) (R refers to the droplet radius, Δr > 0 is the droplet radius extension value), so that only the next step is to deal with the small area where the droplets are located. The specific steps are shown as Figure 8. Figure 9 shows the steps of droplet image preprocessing.

### 4.2. Segmentation of Droplet Images

Image segmentation is the technique and process of segmenting an image into a number of specific regions with unique properties and extracting the target of interest. It is a crucial step from image processing to image analysis. After the previous preprocessing, a small range of regions containing only droplets can be obtained, which reduces the interference factor of the subsequent segmentation of the droplet image and simplifies the difficulty of droplet contour extraction.

The existing image segmentation methods are mainly divided into the following categories: threshold-based segmentation methods, region-based segmentation methods, edge-based segmentation methods, and segmentation methods based on specific theories.

Based on the advantages of the Canny algorithm and the characteristics of the droplet image, the Canny operator in the direction operator is used to detect the edge. The direction operator is based on the differentiation in a particular direction to detect the edge. In the following three standard senses, the Canny operator is optimal for step edges that are affected by white noise.

(1) Testing standards: no important edges are lost, and there should be no false edges.

(2) Positioning criteria: the deviation between the actual edge and the detected edge position is the minimum.

(3) Single response criteria: reduce multiple responses to a single edge response.

**Figure 10:**
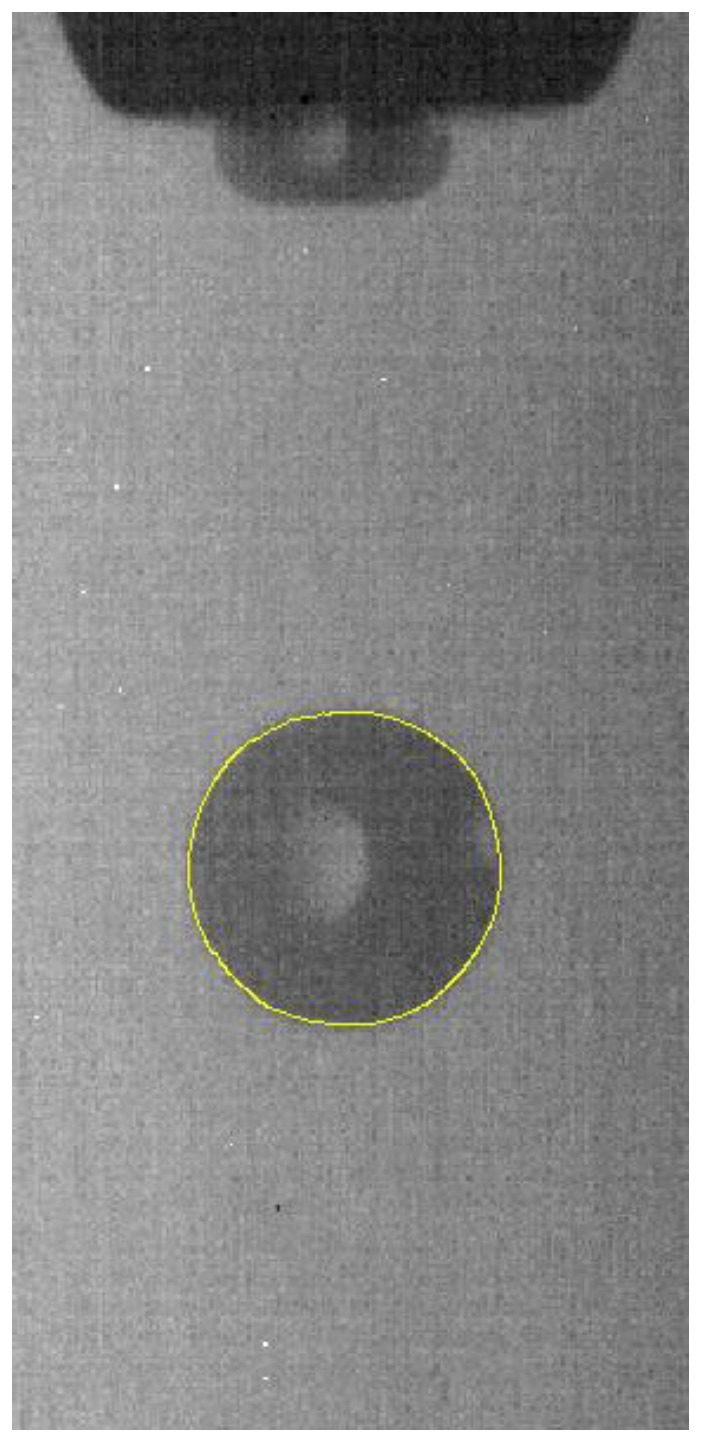
Droplet contour extraction (sub-pixel accuracy).

The edge detection is performed by using the canny operator of Figure 9 to obtain the sub-pixel outline of the droplet, as shown in Figure 10.

### 4.3. Circle Fitting of the Droplet Profile

**Figure 11:**
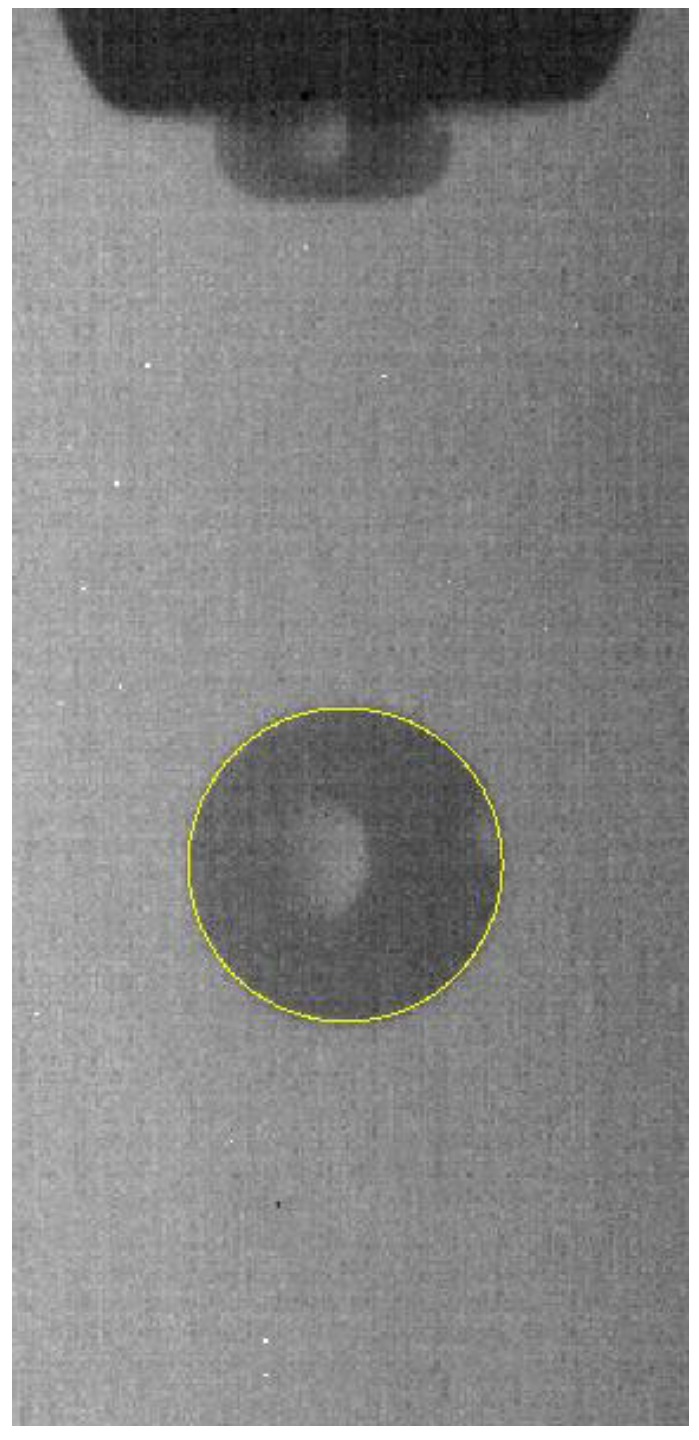
Circle fitting of the droplet profile (sub-pixel accuracy).

Since the image was acquired under steady-state conditions, the droplets gradually became stable during the flight until a standard sphere was formed (assuming the droplets were flying far enough). Therefore, the outline of the droplets in the steady state is approximately circular. If the contour of the droplet extracted in Section 4.2 has a roundness of 0.98 or more, it can be considered that the droplet under this condition is in a stable state, and that its morphology can be approximated as a standard sphere. However, due to the presence of the reflective surface of the droplet and the presence of salt-and-pepper noise, the extracted edges are not smooth. In order to facilitate the calculation of the liquid volume and the accuracy of the edge extraction, the shape of the sub-pixel edge obtained above is fitted. A circle-fit image of the droplet profile as shown in Figure 11 was obtained.

## 5. Measurement of Micro-Drops

### 5.1. The Volume of the Drops

As shown in Figure 11, the fitted curve is an accurate circle; the microdroplet can be described as a sphere. For a spherical model, its volume derives directly from the radius of the two-dimensional contour profile. In this study, the droplet volume is calculated by the radius, the estimated volume is

(1) $$V=\frac{4}{3}\pi R^{3}$$

**Figure 12:**
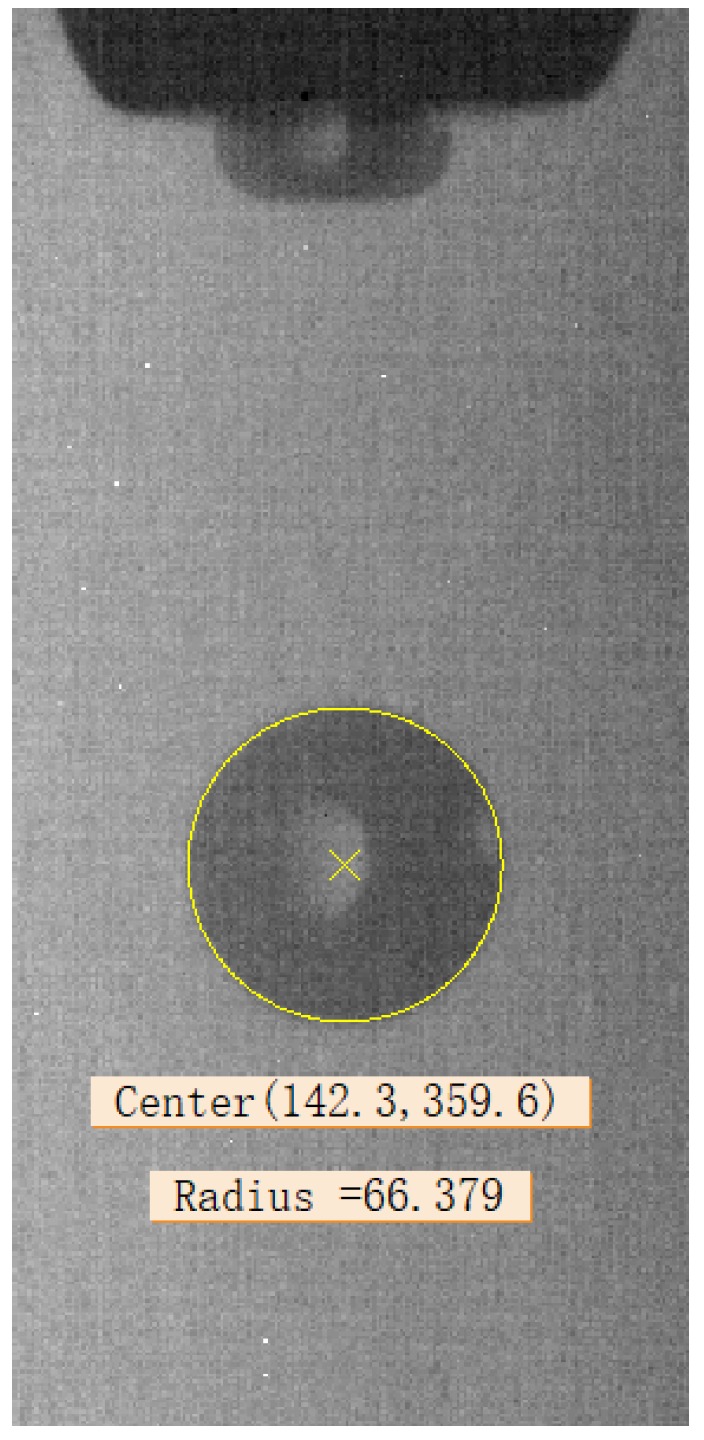
Center coordinates and radius of the droplet (unit: pixel).

According to the results of the circle fitting in Section 4.3, we can easily get the pixel coordinates and radius of the droplet. In this study, one pixel corresponds to ~2.01 μm. The obtained center coordinates and radius are shown in Figure 12.

Therefore, the diameter and volume of the droplet under this condition can be calculated as

*D* = 66.379 × 2.01 × 2 ≈ 266.8 µm

*V* = (4/3) × 3.14 × (66.379 × 2.01)^3^ × 10^−3^ ≈ 9944 pL

The canny algorithm is used for droplet contour extraction, which achieves the sub-pixel accuracy, then the radius deviation value is approximately ±1 pixel (1 pixel = 2.01 μm). Compared with barycentric coordinates and center coordinates extracted from the fitting circle, the coordinate deviation value is approximately ±3 pixels (1 pixel = 2.01 μm). Then, the error range of radius is ±1.5% and the error range of center coordinate is ±2.26%. These shows that the errors were nearly all less than ±3%.

### 5.2. The Speed of the Drops

The microdroplet speed is calculated by varying trigger delay time of CCD which is synchronized in regard to the stimulating signal for jetting. The droplet speed can be calculated from the two CCD camera images with the trigger delay time of CCD. The analyzed results need to be converted to distance in micrometers in order to calculate the speed of a droplet. Therefore, the velocity of the droplet can be calculated according to the following formula.

(2) $$\nu =\frac{p_{y2}-p_{y1}}{t_{2}-t_{1}}\times \mu m/\mathrm{pixel}$$

where, *p_y_* is pixel location of the droplet’s center in *y* direction and *t*_1_ and *t*_2_ are the respective trigger delay times according to the two CCD images.

In order to calculate the velocity change of the process in which droplets are ejected from the nozzle until a stable sphere is formed in the air, the steps to calculate the velocity of the liquid at different times are as follows.

**Figure 13:**
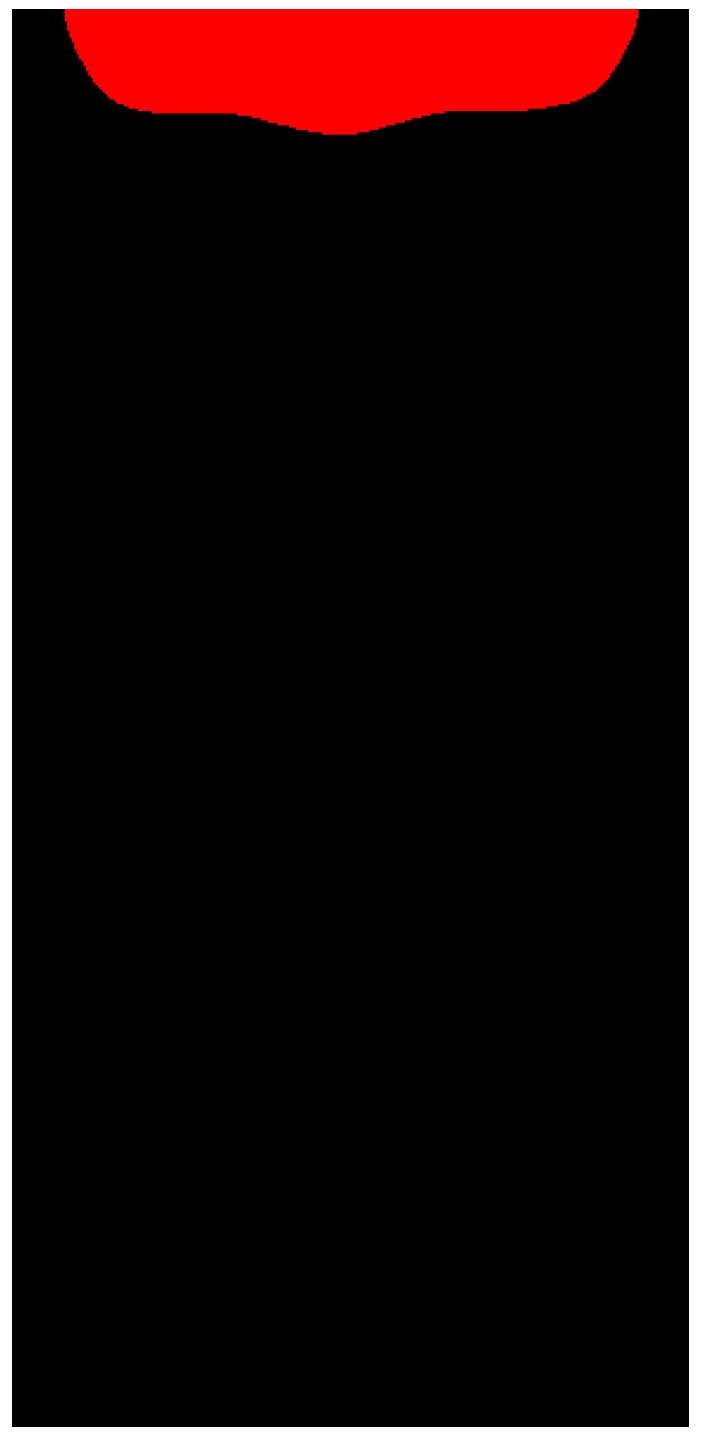
The area where the nozzle is located when there is no injection.

**Figure 14:**
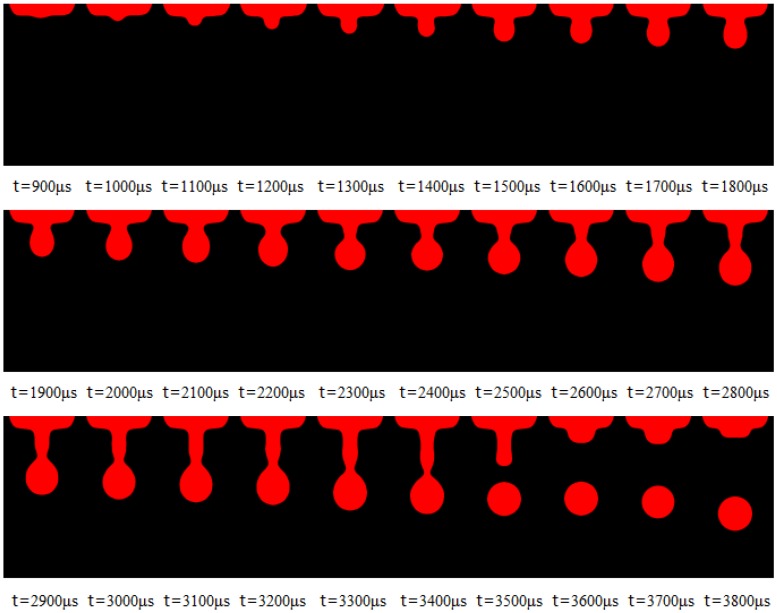
The area where the nozzle and droplet are located at different times.

(a) Collecting an image at the nozzle where no droplets are generated and obtaining the area where the nozzle is located by image processing, the red area as shown in Figure 13 at *t* = 900 μs.

(b) Collecting images of different time delays and obtaining the area of the nozzle and liquid through image processing, the extracted area is shown in the red area in Figure 14.

It should be noted that the images acquired in steps (a) and (b) need to be in the same lighting environment and the image processing parameters should be consistent.

**Figure 15:**
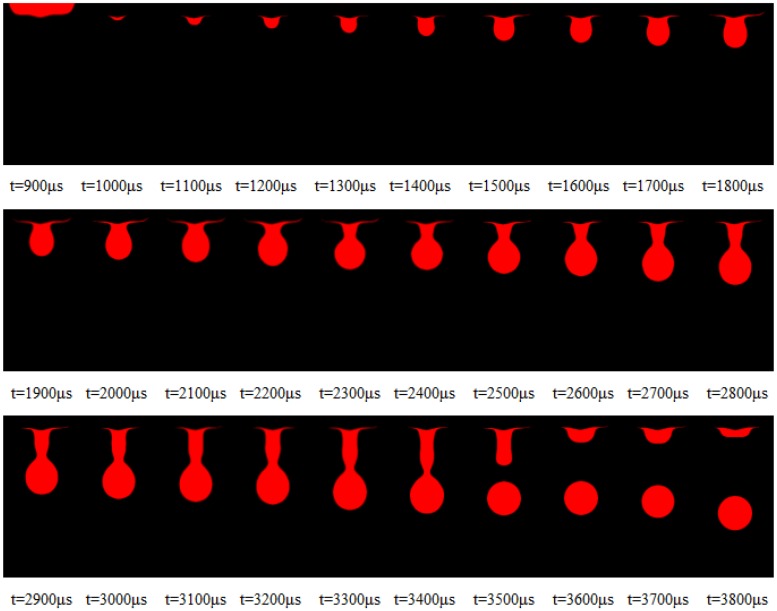
The shape of the droplets outside the nozzle at different times.

(c) Subtracting the region obtained in the step (b) and the region obtained in (a), respectively. Obtaining a region of the liquid outside the nozzle at different times, the results obtained after the regional subtraction are shown in Figure 15.

(d) Calculating the barycentric coordinates of each region by the operator in HALCON, the results are shown in Table 3.

When *t* = 3500 μs, there are two barycentric coordinates and, meanwhile, only one droplet is formed. When calculating the velocity of the droplet at this moment, the generated droplet and the liquid column below the nozzle are regarded as a whole; the reference point is calculated using the overall center of barycentric coordinate as the velocity.

(e) By substituting the barycentric coordinates obtained in step (d) into formula (2), the velocity of the liquid at different time delays can be obtained, as shown in Table 3.

**Figure 16:**
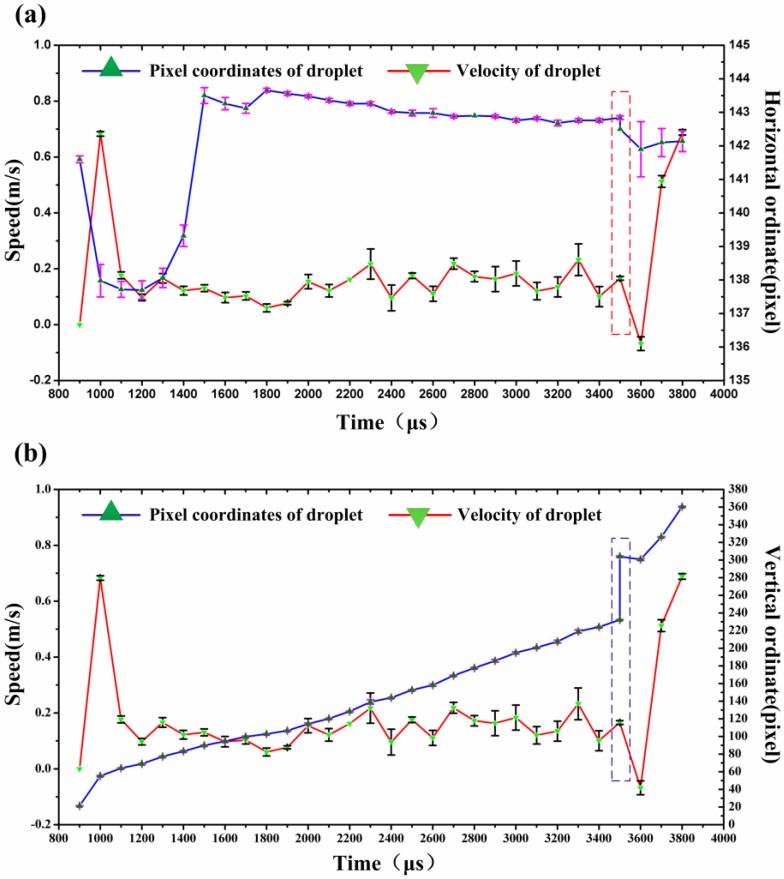
The speed and ordinate of the microdroplet at different times. (**a**) speed and horizontal ordinate VS time, (**b**) speed and vertical ordinate VS time.

According to Table 3, a line chart of average velocity, average abscissa, and ordinate varying with time can be plotted. In addition, in order to reflect the droplet barycentric coordinates (pixel coordinates, including horizontal and longitudinal coordinates) and the fluctuation size of velocity at each moment more clearly, the error bars of velocity, horizontal, and longitudinal coordinates are obtained according to Table 3 and are shown in Figure 16. The technique has higher accuracy compared with the method of calculating velocity with the microdroplet terminal coordinate as the reference point.

Figure 16 describes the variation relationship between droplet velocity and coordinates over time. The velocity of the droplet just ejected from the nozzle is relatively large, reaching ~0.68 m/s, then rapidly decrease under the influence of the viscous resistance and surface tension of the solution and reaches a relatively stable speed (0.18 m/s). After that, the liquid column is necked and fractured to form a droplet, and the velocity of the process is gradually reduced to zero and accompanied by the rebound phenomenon of the droplet. This is the reason why the velocity has a negative value. Finally, the droplets are in flight. According to Figure 16, the conclusions can be drawn as follows.

(1) The variation trend of velocity is basically consistent with the formation process of droplets. That is, in the initial stage, the droplets are rapidly reduced by a larger velocity, and then enter the necking and breaking stage at a relatively stable velocity. Finally, the broken droplets are in flight.

(2) The variation trend of droplet velocity–time curve in five groups is approximately the same, which indicates that the jetting condition is stable under the setting parameters (see Supplementary Material).

(3) During the formation of the droplet, the ratio of the maximum offset of the droplet in the horizontal direction (taking the central axis of the nozzle as the reference) to the total height of the drop is ~1.6%, which indicates that the nozzle is absence from the disturbance of surrounding airflow. Therefore, the influence is almost negligible.

In addition, Figure 16 also shows error bars of velocity and the horizontal and longitudinal coordinates of the droplets at each moment.

Horizontal coordinate error bar: in the process of formation and flight of droplets, there is a tendency of “small middle and big ends”. The reason is that the droplets are greatly influenced by the accumulation of liquid from the end surface of the nozzles when the droplets are just ejected from the nozzles. Since then, as the droplets continue to stretch, the influence of the accumulated fluid is getting smaller and smaller, and the value is kept within a smaller range. At approximately *t* = 3500 μs, one droplet is generated. At this time, the droplet is out of the binding of the liquid column, and is more susceptible to the disturbance of the airflow, and its value is slightly increased. However, the droplets in flight gradually achieve a balance under the coaction of kinetic energy, gravitational potential energy, surface free energy and heat generated from air resistance. Thus, the error bar value is getting smaller and smaller. Until *t* = 3800 μs, the relative standard deviation of the abscissa is 0.22% (standard deviation/average value), indicating that the position oscillation of the droplet in the horizontal direction is very small at this moment.

Longitudinal coordinate error bar: during the entire injection process, the longitudinal coordinate error bar of the droplet is maintained in a small range, which indicates that the droplet position in vertical direction is almost constant at the same delay time. This is another illustration for demonstrating jetting stability.

Velocity error bar: there is a great fluctuation approximately *t* = 3500 μs. At this moment, the velocity of the droplet reveals a significant change (reduces to zero or even a negative velocity) because the rebound phenomenon occurs after the droplet breaks. After that, the droplets in flight gradually achieve a balance in the mutual conversion of various kinds of energy. The shape of droplets is increasingly closer to the standard sphere; meanwhile, the velocity error bar will decrease rapidly. When *t* = 3800 μs, the relative standard deviation of the droplet velocity is reduced to 1.4%, which indicates that the state of the droplet is very stable at this moment.

In summary, just at *t* = 3800 μs, the shape of the droplet and the jetting behavior have reached to a stable state. Then, the working position of the substrate can be set near the height point at this moment, so as to fabricate a good quality pattern on the substrate.

### 5.3. Microsolder Ball Volume Measurement

**Figure 17:**
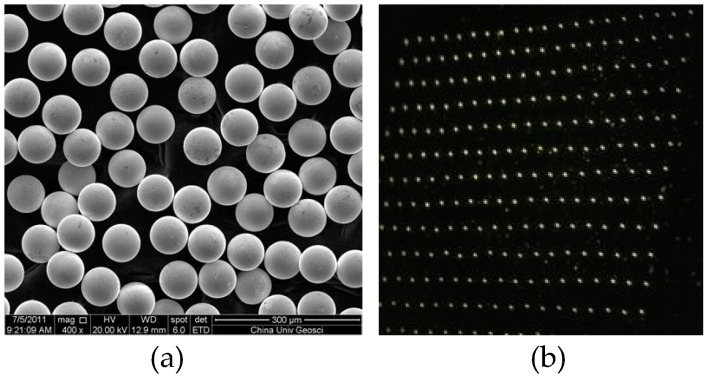
(**a**) Microsolder balls in silicone oil, (**b**) microsolder balls array on stainless steel plate.

**Figure 18:**
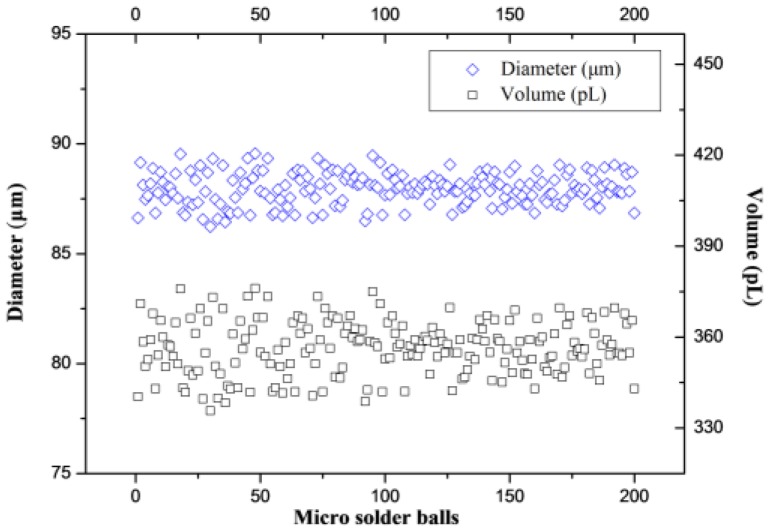
Measured diameters and volumes of 200 microsolder balls.

The pneumatically diaphragm-driven actuator is also applied to generate microsolder balls. The solder brand is 63/37, with 63% tin and 37% lead. The melting point is 183 °C. At 200 °C and the viscosity and surface tension are 2.88 mPa·s and 0.435 N/m. Figure 17a shows microsolder balls in silicone oil and Figure 17b shows microsolder balls array (20 × 20) on stainless steel plate. The detailed volume data of 200 microsolder balls in Figure 17a are plotted in Figure 18. The average diameter is 87.96 μm, the standard deviation is 0.7265 μm, and the relative standard deviation is 0.83%. The average volume is 356.36 pL, the mean standard deviation is 8.8218 pL, and the relative standard deviation is 2.5%.

## 6. Conclusions

This paper proposed a novel in situ image acquisition and measurement of microdroplets based on delay triggering. Using a high-speed CCD camera, a motion control card, a driving circuit, a time delay triggering circuit, and a light source, an imaging system is built to obtain the sequential images of microdroplets generated through the pneumatically diaphragm-driven actuator. By adjusting the delay time, the stable microdroplet fabrication process can be captured. The image processing methods have been adapted to measure the coordinates, diameters and speeds of the microdroplets real-time. For glycerin/water (60:40, mass ratio), under the given condition, the average of diameter and volume are 266.8 μm and 9944 pL, respectively. The maximum average velocity of the microdroplets is 0.689 m/s. Finally, the pneumatically diaphragm-driven actuator is applied to generate microsolder balls. The results show the system processes good reproducibility. The average diameter is 87.96 μm, the standard deviation is 0.7265 μm, and the relative standard deviation is 0.83%. The in situ imaging system employs two control signals to stimulate the microdroplet generator and the CCD camera and is absence of instantaneous high intensity light. Hence, the system can avoid the desynchronization problem of signals which control the strobe LED light source and the camera in previous techniques. The system with low cost and simple configuration is especially applicable to capture and analyze the flight process of microdroplets involving fairly large sizes (~200 μm) moving at low speed (~1 m/s). This technology is a reliable and cost-effective for acquiring and measuring microdroplets.

## Figures and Tables

**Table 1:** Image acquisition system of microdroplets based on delay triggering and their features.

Ref.	Printhead Type	Jetting Material	Number of Signals	Control Object by Trigger Signals		
[5]	Trident printhead by piezo transducer	Glycerin and water mixture in the weight ratio 48/52	3	Trident printhead by piezo transducer	Pulsed Cu vapor laser	CCD camera
[6]	Xaar 126-200 print head with a linear array of 50 µm diameter nozzles	Semi-transparent UV-curable ink	3	Print head	Flash source	Camera
[7]	Piezo inkjet printhead	Nanoparticles dissolved in DI (deionized) water	3	Piezo inkjet printhead	Strobe LED light	CCD camera
[8]	Piezo inkjet printhead	Liquid crystal	3	Piezo inkjet printhead	Stroboscopic light	CCD camera
[9]	Piezoelectric sleeve transducer printhead	Glycerol and water mixture	3	Piezoelectric sleeve transducer printhead	Strobe LED light	CCD camera
[10]	Piezo inkjet printhead	Water, DMSO, NMP, Acetonitrile, Dichloromethane, Methanol	3	Piezo inkjet printhead	Strobe LED light	CCD camera
Proposed article	Pneumatically diaphragm-driven actuator	Glycerin and water mixture (60/40, mass ratio)	2	Pneumatically diaphragm-driven actuator		CCD camera

**Table 2:** The specifications of Volpi Intralux Model 4000-1.

Illumination Intensity at Light Guide Receptacle	Uniformly Illuminated Light Guide Diameter	Rated Lamp Life	Color Temperature	Light Adjustment	Max Housing Temp	Lamp-EPV
320,000 ftc	10 mm	1000 hrs	3150 °K, constant	Crescent shaped diaphragm	100 °K	Halogen reflector, 14.5 V/90 W

**Table 3:** Average abscissa, ordinate, and velocity of droplets at different times and their corresponding standard deviations.

Time (μs)	Average Horizontal Coordinate (pixel)	Horizontal Coordinate Standard Deviation	Average Vertical Coordinate (pixel)	Vertical Coordinate Standard Deviation	Average Velocity (m/s)	Velocity Standard Deviation
900	141.6	0.100	21.3	0.134	0	0
1000	138.0	0.487	55.3	0.415	0.683	0.008
1100	137.7	0.239	64.1	0.383	0.177	0.012
1200	137.7	0.274	69.0	0.503	0.097	0.012
1300	138.1	0.288	77.2	0.546	0.166	0.017
1400	139.3	0.319	83.3	0.711	0.122	0.016
1500	143.5	0.235	89.8	0.460	0.131	0.012
1600	143.3	0.182	94.6	0.546	0.097	0.018
1700	143.1	0.148	99.8	0.502	0.103	0.014
1800	143.7	0.055	102.7	0.555	0.060	0.014
1900	143.6	0.055	106.6	0.327	0.077	0.006
2000	143.5	0.045	114.3	1.122	0.154	0.026
2100	143.4	0.055	120.3	0.349	0.122	0.022
2200	143.3	0.055	128.4	0.349	0.162	0
2300	143.3	0.055	139.1	2.472	0.217	0.054
2400	143.0	0.045	143.9	0.329	0.096	0.046
2500	143.0	0.084	152.7	0.409	0.176	0.010
2600	143.0	0.130	158.2	1.242	0.111	0.027
2700	142.9	0.045	169.0	0.365	0.218	0.020
2800	142.9	0	177.6	0.785	0.172	0.019
2900	142.9	0.045	185.7	1.447	0.163	0.045
3000	142.8	0.055	194.9	1.158	0.183	0.045
3100	142.8	0.045	200.8	0.404	0.120	0.031
3200	142.7	0.084	207.6	1.568	0.135	0.036
3300	142.8	0.055	219.0	1.816	0.233	0.057
3400	142.8	0.055	224.1	0.377	0.101	0.036
3500	142.8	0.055	232.3	0.230	0.166	0.007
3500	142.5	0	304.1	0.518	0.166	0.007
3600	141.9	0.822	300.7	1.055	−0.068	0.025
3700	142.1	0.418	326.2	0.321	0.513	0.021
3800	142.1	0.313	360.4	0.522	0.689	0.010

## Supplementary Materials

The following are available online at https://www.mdpi.com/2072-666X/10/2/148/s1.
