# Supplementary material for: In Situ Image Acquisition and Measurement of Microdroplets Based on Delay Triggering
Source: Micromachines (Basel). 2019 Feb 22;10(2):148. doi: 10.3390/mi10020148 (PMC6412821; doi:10.3390/mi10020148)

Supplementary Materials: In Situ Image Acquisition and Measurement of Microdroplets Based on Delay Triggering

Xuefeng Chang, Kang Zheng, Dan Xie *, Xiayun Shu *, Keyu Xu, Wenhuan Chen, Bo Li and Changjian Wu

The supplementary material consists of five sets of time-series diagrams (original images) of droplet formation and flight process based on the time-delay acquisition system and the final processing renderings, and a line chart of droplet coordinates and velocity as a function of time.

Set 1:

(1) Original images:

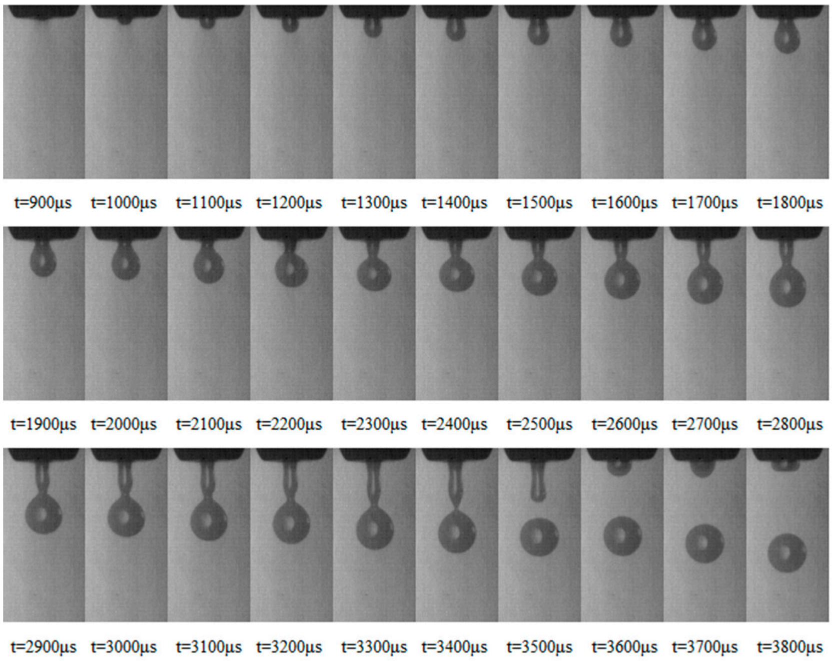

(2) Processed images:

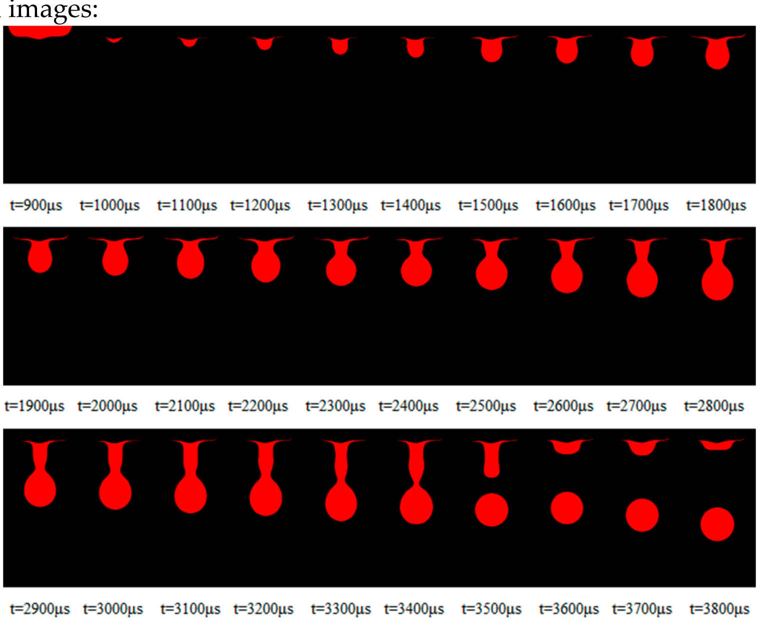

(3) Center of gravity coordinates of liquid (drop) at different times (unit: pixel)

**Time**	900μs	1000μs	1100μs	1200μs	1300μs
**Coordinate**	(141.6,21.5)	(138.2,56.0)	(137.9,63.9)	(137.6,68.2)	(138.3,77.9)
**Time**	1400μs	1500μs	1600μs	1700μs	1800μs
**Coordinate**	(139.1,83.4)	(143.9,89.2)	(143.5,94.9)	(143.3,99.6)	(143.7,103.1)
**Time**	1900μs	2000μs	2100μs	2200μs	2300μs
**Coordinate**	(143.6,106.8)	(143.5,113.8)	(143.4,120.6)	(143.3,128.7)	(143.3,136.5)
**Time**	2400μs	2500μs	2600μs	2700μs	2800μs
**Coordinate**	(143.1,143.7)	(143.1,152.3)	(143.1,159.0)	(142.9,169.4)	(142.9,176.7)
**Time**	2900μs	3000μs	3100μs	3200μs	3300μs
**Coordinate**	(142.9,187.9)	(142.8,194.3)	(142.9,201.2)	(142.8,207.0)	(142.8,216.7)
**Time**	3400μs	3500μs	3500μs	3600μs	3700μs
**Coordinate**	(142.8,223.7)	(142.9,232.5)	(142.5,304.3)	(142.5,299.5)	(142.5,326.5)
**Time**	3800μs				
**Coordinate**	(142.0,361.0)				

(4) Velocity of liquid (drop) at different times (unit: m/s):

**Time**	900μs	1000μs	1100μs	1200μs	1300μs	1400μs
**Speed(m/s)**	0	0.695	0.159	0.086	0.194	0.111
**Time**	1500μs	1600μs	1700μs	1800μs	1900μs	2000μs
**Speed(m/s)**	0.116	0.116	0.093	0.072	0.073	0.141
**Time**	2100μs	2200μs	2300μs	2400μs	2500μs	2600μs
**Speed(m/s)**	0.138	0.162	0.158	0.144	0.173	0.135
**Time**	2700μs	2800μs	2900μs	3000μs	3100μs	3200μs
**Speed(m/s)**	0.209	0.146	0.225	0.219	0.139	0.117
**Time**	3300μs	3400μs	3500μs	3600μs	3700μs	3800μs
**Speed(m/s)**	0.141	0.177	0.177	-0.096	0.543	0.693

(5) Coordinate-time and speed-time line chart:

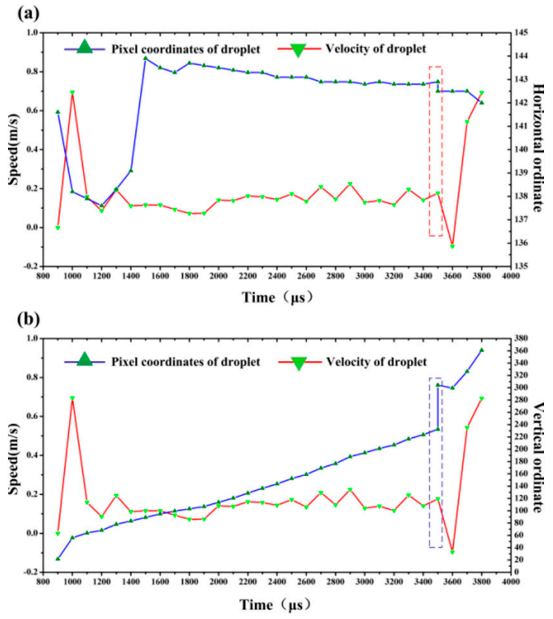

Set 2:

(1) Original images:

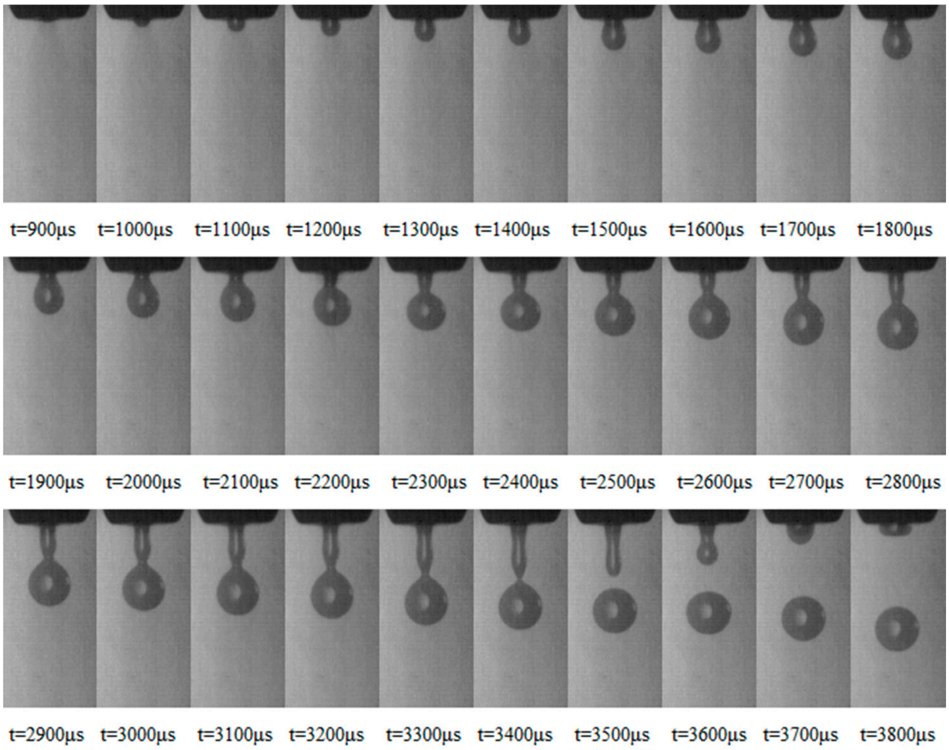

(2) Processed images:

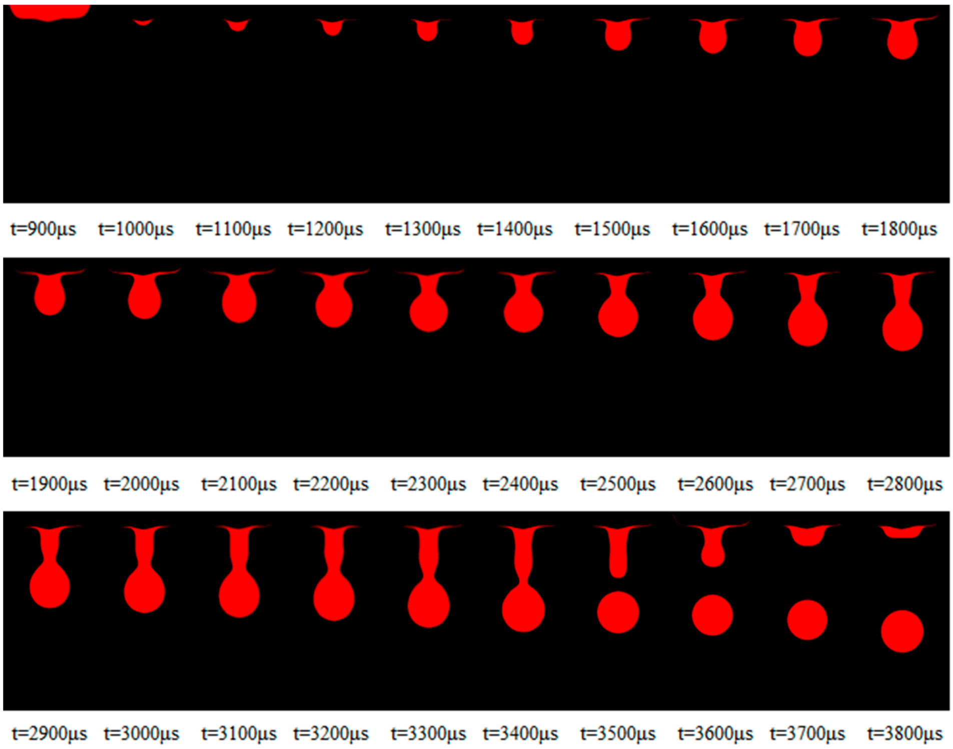

(3) Center of gravity coordinates of liquid (drop) at different times (unit: pixel)

**Time**	900μs	1000μs	1100μs	1200μs	1300μs
**Coordinate**	(141.7,21.4)	(138.7,55.2)	(137.4,64.2)	(137.9,69.4)	(138.3,77.7)
**Time**	1400μs	1500μs	1600μs	1700μs	1800μs

**Coordinate**	(139.1,83.3)	(143.5,89.8)	(143.2,95.3)	(143.2,99.4)	(143.6,103.2)
**Time**	1900μs	2000μs	2100μs	2200μs	2300μs
**Coordinate**	(143.5,106.7)	(143.4,113.7)	(143.3,120.5)	(143.2,128.6)	(143.2,141.4)
**Time**	2400μs	2500μs	2600μs	2700μs	2800μs
**Coordinate**	(143.0,143.7)	(142.9,153.2)	(143.0,158.6)	(142.9,169.3)	(142.9,178.3)
**Time**	2900μs	3000μs	3100μs	3200μs	3300μs
**Coordinate**	(142.8,184.9)	(142.7,194.3)	(142.8,201.1)	(142.6,207.1)	(142.8,218.9)
**Time**	3400μs	3500μs	3500μs	3600μs	3700μs
**Coordinate**	(142.8,224.2)	(142.8,232.3)	(142.5,303.5)	(141.0,299.9)	(141.5,325.8)
**Time**	3800μs				
**Coordinate**	(141.8,360.7)				

(4) Velocity of liquid (drop) at different times (unit: m/s):

**Time**	900μs	1000μs	1100μs	1200μs	1300μs	1400μs
**Speed(m/s)**	0	0.678	0.181	0.106	0.166	0.114
**Time**	1500μs	1600μs	1700μs	1800μs	1900μs	2000μs
**Speed(m/s)**	0.129	0.110	0.083	0.077	0.070	0.140
**Time**	2100μs	2200μs	2300μs	2400μs	2500μs	2600μs
**Speed(m/s)**	0.138	0.162	0.258	0.045	0.192	0.108
**Time**	2700μs	2800μs	2900μs	3000μs	3100μs	3200μs
**Speed(m/s)**	0.215	0.180	0.133	0.188	0.138	0.120
**Time**	3300μs	3400μs	3500μs	3600μs	3700μs	3800μs
**Speed(m/s)**	0.107	0.163	0.163	-0.072	0.521	0.701

(5) Coordinate-time and speed-time line chart:

(a)

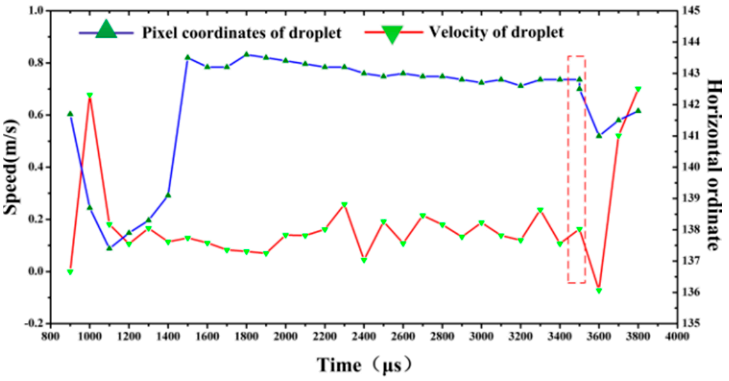

(b)

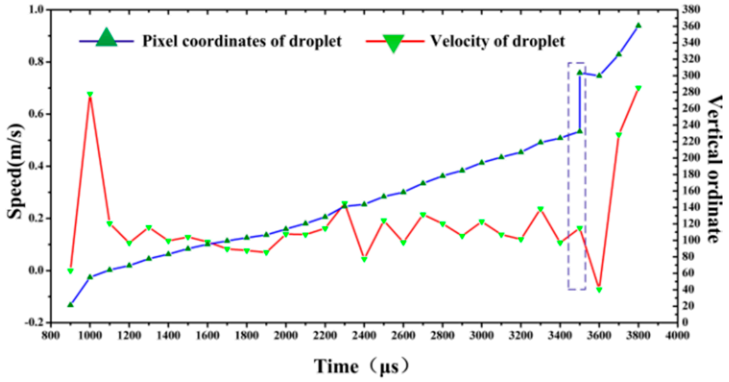

Set 3:

(1) Original images:

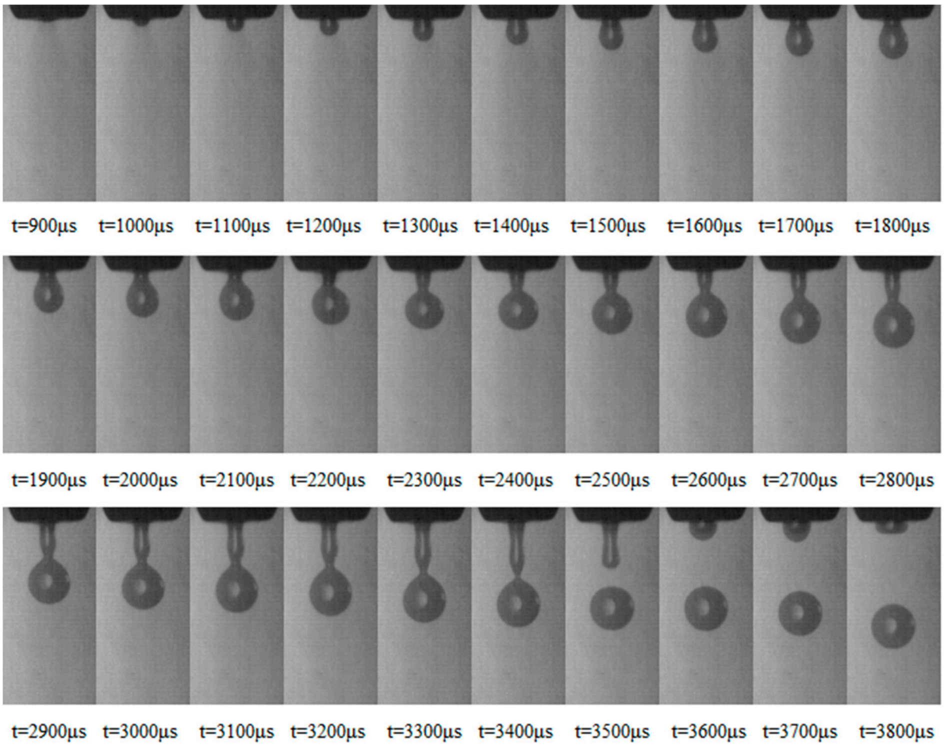

(2) Processed images:

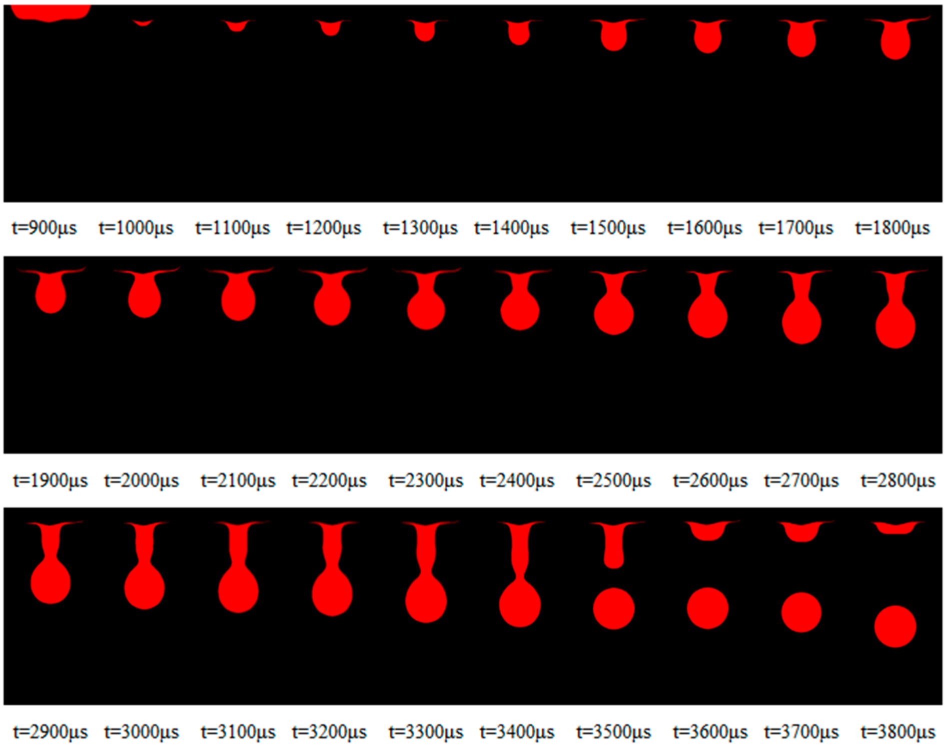

(3) Center of gravity coordinates of liquid (drop) at different times (unit: pixel)

**Time**	900µs	1000µs	1100µs	1200µs	1300µs
**Coordinate**	(141.7,21.4)	(137.5,55.0)	(138.0,63.6)	(137.7,69.3)	(138.0,77.1)
**Time**	1400µs	1500µs	1600µs	1700µs	1800µs

**Coordinate**	(139.5,83.2)	(143.4,89.8)	(143.0,94.8)	(142.9,100.6)	(143.7,102.8)
**Time**	1900μs	2000μs	2100μs	2200μs	2300μs
**Coordinate**	(143.5,106.7)	(143.5,115.8)	(143.3,120.5)	(143.2,128.6)	(143.2,136.4)
**Time**	2400μs	2500μs	2600μs	2700μs	2800μs
**Coordinate**	(143.0,143.7)	(143.0,152.3)	(142.8,158.9)	(142.8,169.2)	(142.9,177.6)
**Time**	2900μs	3000μs	3100μs	3200μs	3300μs
**Coordinate**	(142.9,185.3)	(142.7,193.9)	(142.8,201.1)	(142.6,207.1)	(142.8,218.9)
**Time**	3400μs	3500μs	3500μs	3600μs	3700μs
**Coordinate**	(142.7,224.1)	(142.8,232.4)	(142.5,304.5)	(142.5,300.5)	(142.0,326.0)
**Time**	3800μs				
**Coordinate**	(142.3,359.6)				

(4) Velocity of liquid (drop) at different times (unit: m/s):

**Time**	900μs	1000μs	1100μs	1200μs	1300μs	1400μs
**Speed(m/s)**	0	0.675	0.173	0.113	0.158	0.121
**Time**	1500μs	1600μs	1700μs	1800μs	1900μs	2000μs
**Speed(m/s)**	0.134	0.100	0.116	0.045	0.078	0.182
**Time**	2100μs	2200μs	2300μs	2400μs	2500μs	2600μs
**Speed(m/s)**	0.096	0.162	0.158	0.146	0.173	0.133
**Time**	2700μs	2800μs	2900μs	3000μs	3100μs	3200μs
**Speed(m/s)**	0.208	0.169	0.154	0.172	0.146	0.120
**Time**	3300μs	3400μs	3500μs	3600μs	3700μs	3800μs
**Speed(m/s)**	0.105	0.167	0.167	-0.080	0.513	0.675

(5) Coordinate-time and speed-time line chart:

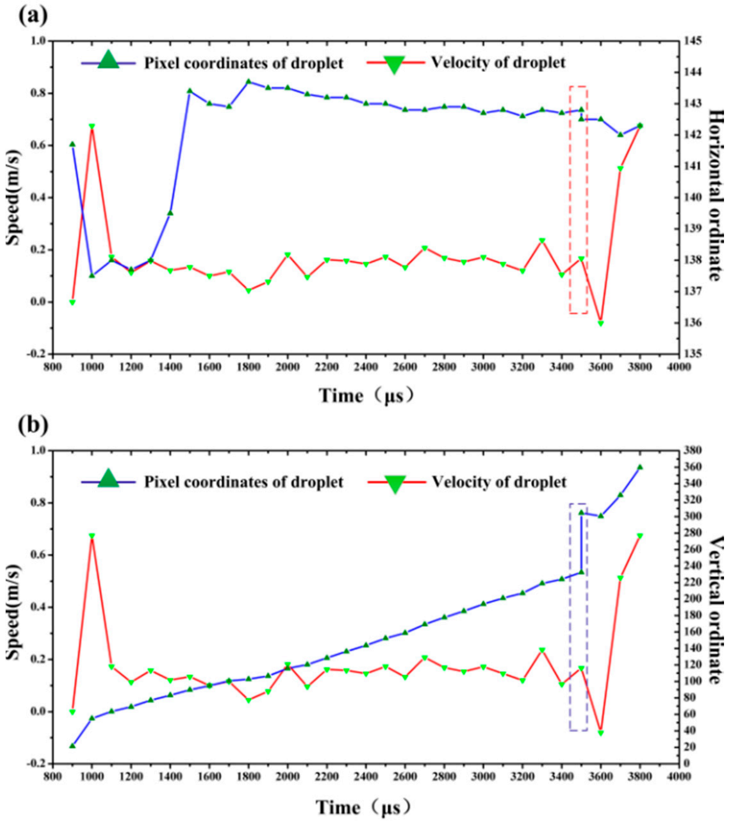

Set 4:

(1) Original images:

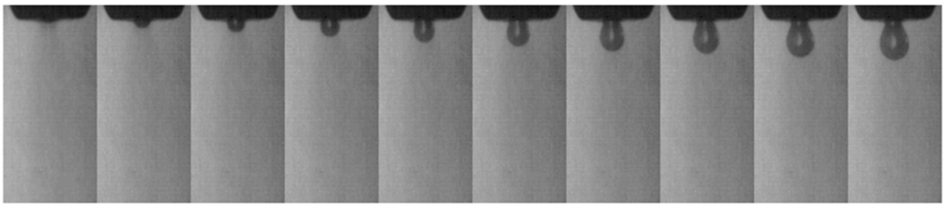

t=900μs t=1000μs t=1100μs t=1200μs t=1300μs t=1400μs t=1500μs t=1600μs t=1700μs t=1800μs

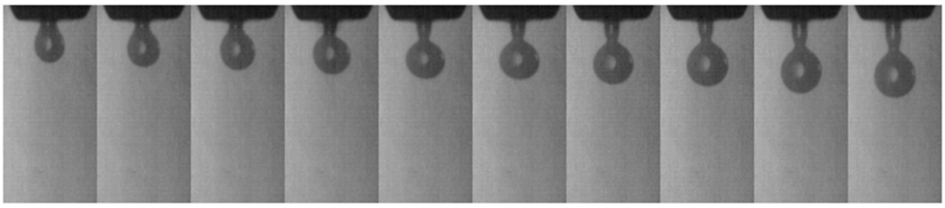

t=1900μs t=2000μs t=2100μs t=2200μs t=2300μs t=2400μs t=2500μs t=2600μs t=2700μs t=2800μs

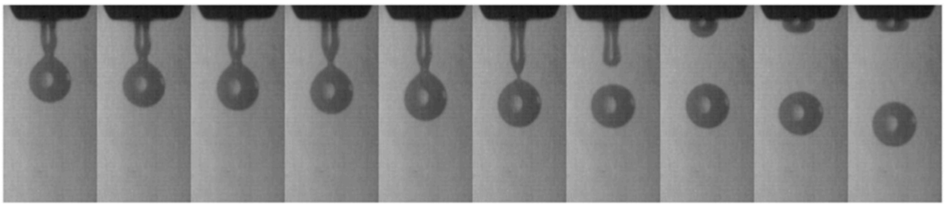

t=2900μs t=3000μs t=3100μs t=3200μs t=3300μs t=3400μs t=3500μs t=3600μs t=3700μs t=3800μs

(2) Processed images:

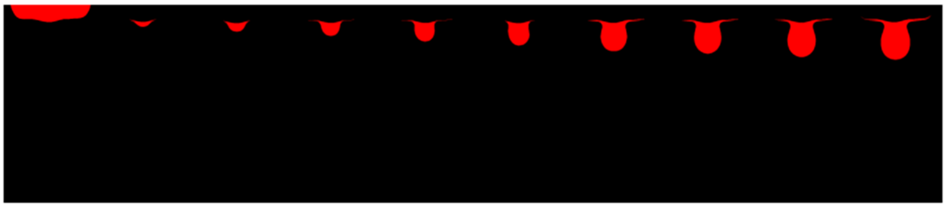

t=900μs t=1000μs t=1100μs t=1200μs t=1300μs t=1400μs t=1500μs t=1600μs t=1700μs t=1800μs

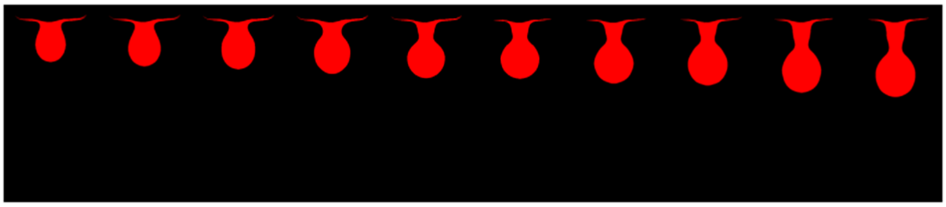

t=1900μs t=2000μs t=2100μs t=2200μs t=2300μs t=2400μs t=2500μs t=2600μs t=2700μs t=2800μs

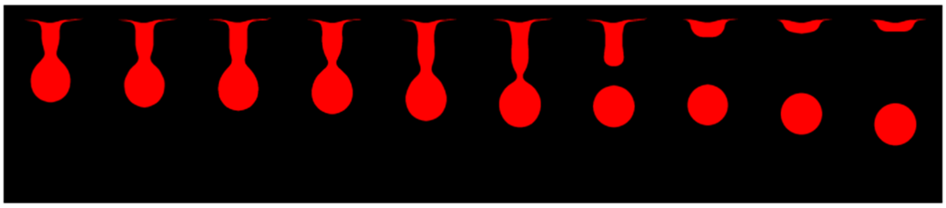

t=2900μs t=3000μs t=3100μs t=3200μs t=3300μs t=3400μs t=3500μs t=3600μs t=3700μs t=3800μs

(3) Center of gravity coordinates of liquid (drop) at different times (unit: pixel):

**Time**	900μs	1000μs	1100μs	1200μs	1300μs
**Coordinate**	(141.5,21.2)	(137.6,55.4)	(137.6,64.3)	(137.3,68.7)	(138.1,76.9)
**Time**	1400μs	1500μs	1600μs	1700μs	1800μs

**Coordinate**	(139.1,84.3)	(143.4,90.5)	(143.3,93.9)	(143.1,99.4)	(143.6,101.8)
**Time**	1900μs	2000μs	2100μs	2200μs	2300μs
**Coordinate**	(143.6,106.0)	(143.5,115.0)	(143.4,119.9)	(143.3,128.0)	(143.3,140.7)
**Time**	2400μs	2500μs	2600μs	2700μs	2800μs
**Coordinate**	(143.0,144.3)	(143.0,152.6)	(143.1,156.0)	(142.9,168.6)	(142.9,177.0)
**Time**	2900μs	3000μs	3100μs	3200μs	3300μs
**Coordinate**	(142.9,186.4)	(142.8,195.1)	(142.8,200.4)	(142.7,206.3)	(142.7,221.8)
**Time**	3400μs	3500μs	3500μs	3600μs	3700μs
**Coordinate**	(142.8,223.9)	(142.9,231.9)	(142.5,304.5)	(142.5,301.5)	(142.0,326.0)
**Time**	3800μs				
**Coordinate**	(142.6,360.4)				

(4) Velocity of liquid (drop) at different times (unit: m/s):

**Time**	900μs	1000μs	1100μs	1200μs	1300μs	1400μs
**Speed(m/s)**	0	0.688	0.178	0.089	0.164	0.149
**Time**	1500μs	1600μs	1700μs	1800μs	1900μs	2000μs
**Speed(m/s)**	0.125	0.069	0.111	0.048	0.085	0.181
**Time**	2100μs	2200μs	2300μs	2400μs	2500μs	2600μs
**Speed(m/s)**	0.098	0.162	0.256	0.072	0.166	0.068
**Time**	2700μs	2800μs	2900μs	3000μs	3100μs	3200μs
**Speed(m/s)**	0.253	0.169	0.190	0.175	0.106	0.119
**Time**	3300μs	3400μs	3500μs	3600μs	3700μs	3800μs
**Speed(m/s)**	0.042	0.161	0.161	-0.060	0.493	0.691

(5) Coordinate-time and speed-time line chart:

(a)

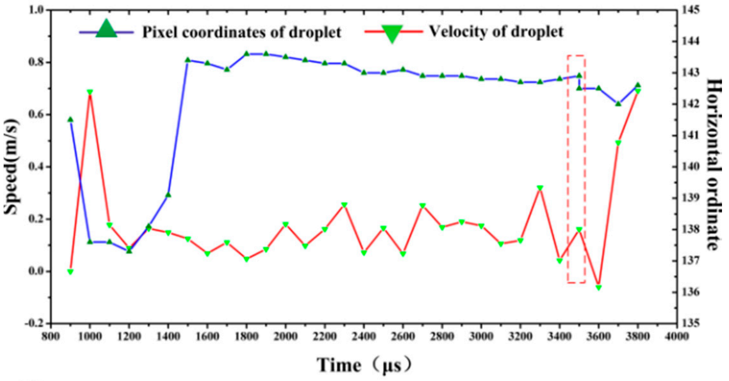

(b)

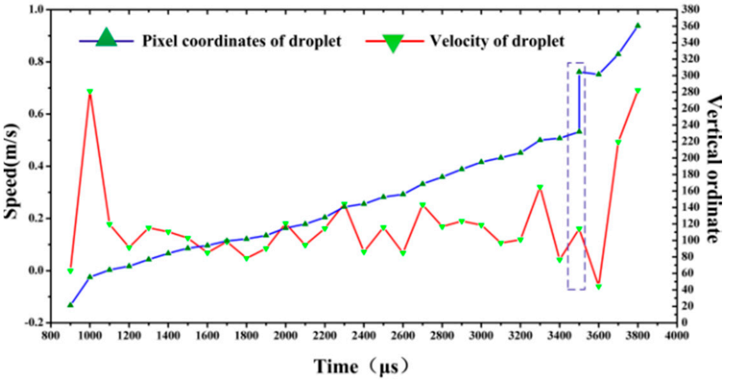

Set 5:

(1) Original images:

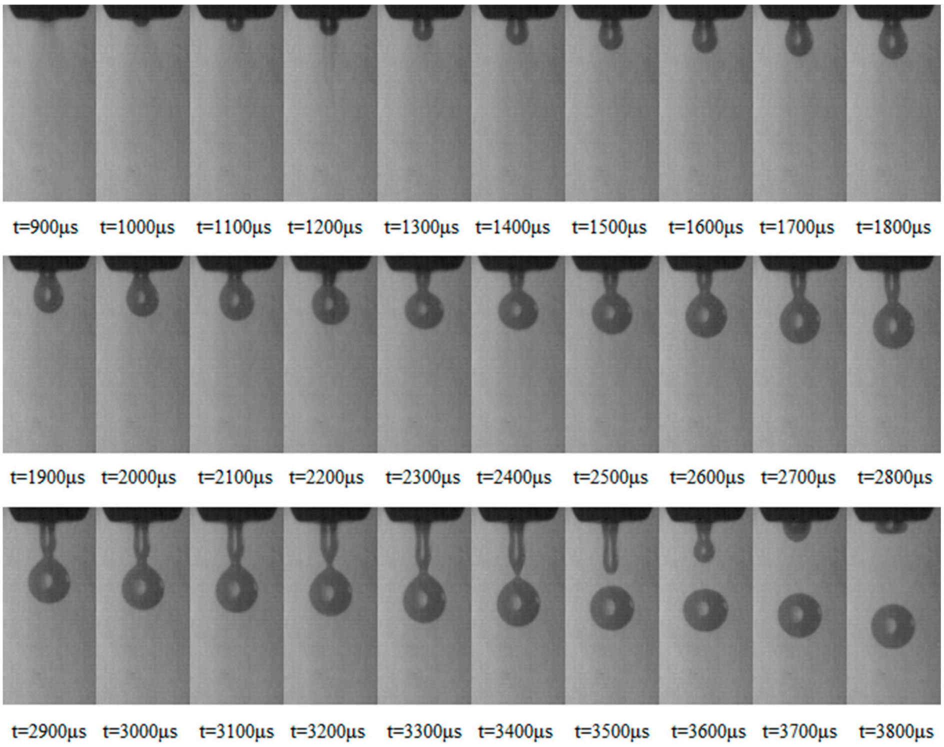

(2) Processed images:

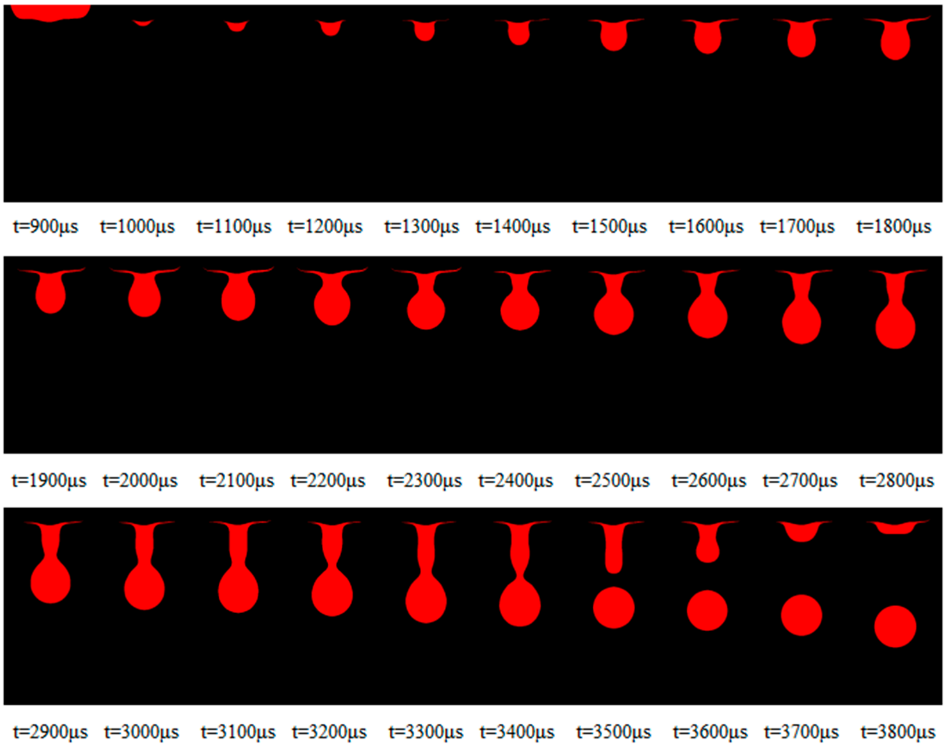

(3) Center of gravity coordinates of liquid (drop) at different times (unit: pixel):

**Time**	900µs	1000µs	1100µs	1200µs	1300µs
**Coordinate**	(141.5,21.2)	(137.9,55.0)	(137.7,64.6)	(138.0,69.2)	(137.6,76.6)
**Time**	1400µs	1500µs	1600µs	1700µs	1800µs

**Coordinate**	(139.8,82.3)	(143.3,89.8)	(143.3,94.3)	(143.1,99.9)	(143.7,102.8)
**Time**	1900µs	2000µs	2100µs	2200µs	2300µs
**Coordinate**	(143.6,106.7)	(143.5,113.0)	(143.4,119.9)	(143.3,128.0)	(143.3,140.7)
**Time**	2400µs	2500µs	2600µs	2700µs	2800µs
**Coordinate**	(143.0,144.3)	(142.9,153.0)	(142.9,158.4)	(142.9,168.7)	(142.9,178.5)
**Time**	2900µs	3000µs	3100µs	3200µs	3300µs
**Coordinate**	(142.9,184.2)	(142.8,196.8)	(142.8,200.4)	(142.7,210.3)	(142.7,218.8)
**Time**	3400µs	3500µs	3500µs	3600µs	3700µs
**Coordinate**	(142.7,224.7)	(142.8,232.2)	(142.5,303.5)	(141.0,302.0)	(142.5,326.5)
**Time**	3800µs				
**Coordinate**	(142.0,360.5)				

(4) Velocity of liquid (drop) at different times (unit: m/s):

**Time**	900µs	1000µs	1100µs	1200µs	1300µs	1400µs
**Speed(m/s)**	0	0.679	0.193	0.093	0.148	0.115
**Time**	1500µs	1600µs	1700µs	1800µs	1900µs	2000µs
**Speed(m/s)**	0.149	0.091	0.112	0.059	0.078	0.127
**Time**	2100µs	2200µs	2300µs	2400µs	2500µs	2600µs
**Speed(m/s)**	0.138	0.162	0.256	0.072	0.174	0.109
**Time**	2700µs	2800µs	2900µs	3000µs	3100µs	3200µs
**Speed(m/s)**	0.207	0.197	0.114	0.253	0.072	0.199
**Time**	3300µs	3400µs	3500µs	3600µs	3700µs	3800µs
**Speed(m/s)**	0.108	0.161	0.161	-0.030	0.493	0.683

(5) Coordinate-time and speed-time line chart:

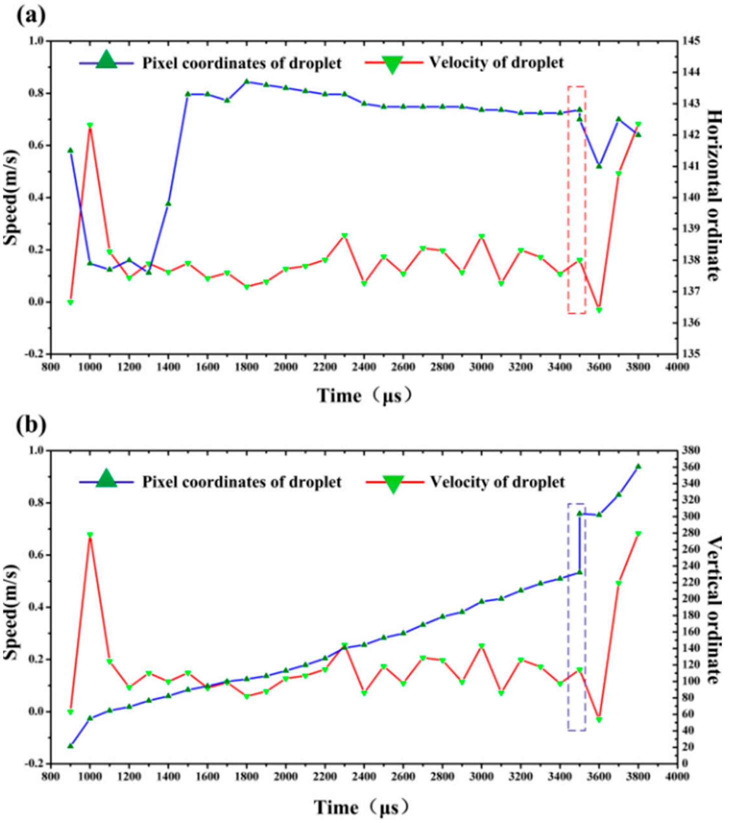

Supplement: Supplementary file 1 [file micromachines-10-00148-s001.pdf]
